# Serum Concentrations of Organochlorine Pesticides and Growth among Russian Boys

**DOI:** 10.1289/ehp.1103743

**Published:** 2011-10-07

**Authors:** Jane S. Burns, Paige L. Williams, Oleg Sergeyev, Susan A. Korrick, Mary M. Lee, Boris Revich, Larisa Altshul, Julie T. Del Prato, Olivier Humblet, Donald G. Patterson, Wayman E. Turner, Mikhail Starovoytov, Russ Hauser

**Affiliations:** 1 Environmental and Occupational Medicine and Epidemiology Program, Department of Environmental Health, and; 2 Department of Biostatistics, Harvard School of Public Health, Boston, Massachusetts, USA; 3 Department of Physical Education and Health, Samara State Medical University, Samara, Russia; 4 Chapaevsk Medical Association, Chapaevsk, Russia; 5 Channing Laboratory, Department of Medicine, Brigham and Women’s Hospital, Harvard Medical School, Boston, Massachusetts, USA; 6 Pediatric Endocrine Division, Departments of Pediatrics and Cell Biology, University of Massachusetts Medical School, Worcester, Massachusetts, USA; 7 Department of Environmental Health, Institute for Forecasting, Russian Academy of Sciences, Moscow, Russia; 8 Environmental Health and Engineering, Inc., Needham, Massachusetts, USA; 9 Exposure, Epidemiology, and Risk Program, Department of Environmental Health, Harvard School of Public Health, Boston, Massachusetts, USA; 10 Division of Immunology and Allergy, Department of Pediatrics, Stanford University, Stanford, California, USA; 11 Centers for Disease Control and Prevention, Atlanta, Georgia, USA; 12 EnviroSolutions Consulting, Inc., Auburn, Georgia, USA; 13 Axys Analytical Solutions, Sidney, British Columbia, Canada; 14 Fluid Management Systems, Boston, Massachusetts, USA; 15 Exponent, Inc., Maynard, Massachusetts, USA; 16 Russian Institute of Nutrition, Moscow, Russia

**Keywords:** BMI, children, DDE, environment, epidemiology, HCB, height, hexachlorocyclohexane, organochlorine pesticides

## Abstract

Background: Limited human data suggest an association of organochlorine pesticides (OCPs) with adverse effects on children’s growth.

Objective: We evaluated the associations of OCPs with longitudinally assessed growth among peripubertal boys from a Russian cohort with high environmental OCP levels.

Methods: A cohort of 499 boys enrolled in the Russian Children’s Study between 2003 and 2005 at 8–9 years of age were followed prospectively for 4 years. At study entry, 350 boys had serum OCPs measured. Physical examinations were conducted at entry and annually. The longitudinal associations of serum OCPs with annual measurements of body mass index (BMI), height, and height velocity were examined by multivariate mixed-effects regression models for repeated measures, controlling for potential confounders.

Results: Among the 350 boys with OCP measurements, median serum hexachlorobenzene (HCB), β-hexachlorocyclohexane (βHCH), and *p*,*p*´-dichlorodiphenyldichloroethylene (*p*,*p*´-DDE) concentrations were 159 ng/g lipid, 168 ng/g lipid, and 287 ng/g lipid, respectively. Age-adjusted BMI and height *z*-scores generally fell within the normal range per World Health Organization standards at entry and during follow-up. However, in adjusted models, boys with higher serum HCB, βHCH, and *p*,*p*´-DDE had significantly lower mean [95% confidence interval (CI)] BMI *z*-scores, by –0.84 (–1.23, –0.46), –1.32 (–1.70, –0.95), and –1.37 (–1.75, –0.98), respectively, for the highest versus lowest quintile. In addition, the highest quintile of *p*,*p*´-DDE was associated with a significantly lower mean (95% CI) height *z*-score, by –0.69 (–1.00, –0.39) than that of the lowest quintile.

Conclusions: Serum OCP concentrations measured at 8–9 years of age were associated with reduced growth, particularly reduced BMI, during the peripubertal period, which may affect attainment of optimal adult body mass and height.

Dichlorodiphenyltrichloroethane (DDT), its metabolite *p*,*p*´-dichlorodiphenyldichloroethylene (*p*,*p*´-DDE), hexachlorobenzene (HCB), and β-hexachlorocyclohexane (βHCH) are organochlorine pesticides (OCPs) that are ubiquitous environmental pollutants, despite being banned (HCB and βHCH) or greatly restricted (DDT) (United Nations Environmental Programme 2009). These lipophilic compounds accumulate in the food chain and have half-lives of years to decades (Longnecker 2005; Wolff et al. 2000). In humans, the most common route of exposure is diet (Darnerud et al. 2006; Marti-Cid et al. 2008). OCPs readily cross the placenta (Carrizo et al. 2006) and concentrate in breast milk (Karmaus et al. 2001; Wolff et al. 2005), thereby leading to infant exposures. Although body burdens of OCPs have decreased over time (Colles et al. 2008; Link et al. 2005), children continue to be exposed.

Children may be especially vulnerable to the effects of endocrine-disrupting OCPs (Eskenazi et al. 2009; Landrigan et al. 2004). Prenatal exposure to *p*,*p*´-DDE and HCB has been associated with reduced birth weight and length, independent of gestational age (Eggesbo et al. 2009; Ribas-Fito et al. 2002; Siddiqui et al. 2003; Weisskopf et al. 2005; Wolff et al. 2007), although estimated associations with postnatal growth have been inconsistent (Eskenazi et al. 2009; Mendez et al. 2011; Smink et al. 2008). Despite concern regarding the effects of DDT and its metabolites on children’s health, including growth (Eskenazi et al. 2009), expanded DDT use is advocated for malaria control (Griffin et al. 2010).

We investigated childhood exposures to OCPs in a cohort of boys in Chapaevsk, Russia, a town highly contaminated with HCB, βHCH, dioxins, and polychlorinated biphenyls (PCBs) from local chemical plants (Ecological Analytical Center 2007; Revich et al. 1999). We report data on the associations of serum OCPs at study entry with serial measures of growth during 4 years of follow-up.

## Methods

*Study population.* The Russian Children’s Study is a prospective cohort study of 499 boys in Chapaevsk, Russia, described in detail elsewhere (Burns et al. 2009). The boys, identified using the townwide health insurance information system, were enrolled at 8 or 9 years of age from 2003 to 2005. Exclusion criteria included being institutionalized or having severe cerebral palsy. OCPs were not measured for the first 144 boys recruited into the study, and five boys with severe chronic illnesses were excluded from the present analysis, leaving 350 boys with OCPs measured. The retention rate was 86% after 4 years. The study was approved by the human studies institutional review boards of the Chapaevsk Medical Association, Harvard School of Public Health, Brigham and Women’s Hospital, and University of Massachusetts Medical School. The parents or guardians signed informed consent forms, and the boys signed assent forms.

At study entry, the boys had a physical examination and blood draw. The mother or guardian completed a nurse-administered health and lifestyle questionnaire (Hauser et al. 2005; Lee et al. 2003) that included birth history, family and child’s medical history, occupational and residential history, household income, and parental education. Birth weight and gestational age were obtained from medical records. A validated Russian Institute of Nutrition semiquantitative food frequency questionnaire was used to ascertain the child’s dietary intake (Martinchik et al. 1998; Rockett et al. 1997).

*Physical examination.* At study entry and annual follow-up visits, a standardized anthropometric examination was performed by a single study investigator (O.S.) per written protocol and without knowledge of the boys’ pesticide levels. Height was measured to the nearest 0.1 cm using a stadiometer. Weight was measured to the nearest 100 g with a balance scale. Age-adjusted *z*-scores were calculated for height and body mass index (BMI; kilograms per square meter) using the World Health Organization (WHO) standards (WHO 2011), and for weight using the Centers for Disease Control and Prevention (CDC) standards (CDC 2009) because WHO standards are unavailable for this age group. Annual height velocity (HV) was calculated for five 1-year intervals (ages 8–13 years) by computing the difference in height between visits, with each boy contributing up to four measurements.

*Blood sample analyses.* Sera from enrollment blood samples were stored at –35°C until shipment on dry ice to the CDC (Atlanta, GA, USA) for organochlorine analysis. The samples, including method blank and quality control samples, were spiked with ^13^C_12_-labeled pesticides, extracted by a C_18_ solid-phase extraction (SPE) followed by a multicolumn automated cleanup and enrichment procedure using either large-volume (Turner et al. 1997) or small-volume (Sjodin et al. 2004) SPE and analyzed using high-resolution mass spectrometry in selective ion monitoring (Barr et al. 2003). Sera were analyzed for dioxin-like compounds [DLCs (polychlorinated dibenzo-*p*-dioxins, dibenzofurans, coplanar PCBs)] and PCBs, and whole blood was analyzed for blood lead levels (BLLs) as described previously (Burns et al. 2009; Hauser et al. 2008; Williams et al. 2010). Lipid-adjusted total 2005 toxic equivalents (TEQs; a measure of toxicity for DLCs) was calculated (Burns et al. 2009).

*Statistical analysis.* We evaluated the associations of serum OCP concentrations measured at 8–9 years of age with the boys’ age-adjusted BMI and height *z*-scores and HV across five study visits (entry and up to four annual follow-up visits) through May 2008. Individual serum OCPs (HCB, βHCH, and *p*,*p*´-DDE) were divided into quintiles, with the lowest quintile as the reference group. We used mixed-effects regression models for repeated measures with an unstructured covariance to examine the associations of OCP quintiles with growth measures. We evaluated univariate associations based on prior literature, fitted a full multivariate model including all covariates with *p* ≤ 0.20, and then reduced it to a core model including covariates with *p* < 0.10 and those required *a priori* for biological interpretability of other covariates. This core model was used for all statistical analyses and included boy’s age, birth weight, and gestational age categories (< 37, 37–42, > 42 weeks); household income categories (< $US175, $175–250, > $250 per month); total calories; percent calories from carbohydrate, fat, and protein; and high (> 5 µg/dL) versus low BLL. In our analyses, statistical significance for main effects and interactions was set at α = 0.05. Tests for trend over OCP levels were performed by modeling quintiles of exposure as a continuous variable. In sensitivity analyses, we adjusted for parental height and weight because these data were available for only 67% (*n* = 236) of fathers and 94% (*n* = 329) of mothers. Our primary models did not adjust for pubertal stage because OCPs may affect pubertal stage and thus be on the causal pathway between OCP exposures and growth. However, we conducted sensitivity analyses adjusting for pubertal stage based on Tanner genitalia staging (Tanner and Whitehouse 1976) (stage 4–5, 2–3, or 1) to confirm OCPs associations with growth. We also performed sensitivity analyses adjusting for quintiles of serum total TEQs, DLCs, and PCBs (non-dioxin-like and mono-*ortho*) because of our prior findings of associations between these compounds and longitudinal growth measures (Burns et al. 2011). We assessed whether the associations between OCPs and growth were modified by age, using an interaction term of continuous age times an indicator of OCP concentrations above the median. In addition, we fitted a joint model including all OCPs simultaneously to evaluate their independent contributions in the context of multiple exposures.

## Results

*Study population and serum OCP concentrations.* Among the 350 boys, rates of prematurity (< 37 weeks) and low birth weight (< 2,500 g) were 8.9% and 5.1%, respectively, similar to U.S. rates (National Vital Statistics Reports 2007). The percentages of calories from dietary protein, fat, and carbohydrate (data not shown) were within age-specific nutritionally appropriate ranges (Food and Nutrition Board 2005). Birth, maternal, and household characteristics are presented in Table 1.

**Table 1:** Descriptive characteristics of the participants of the Russian Children’s Study (*n* = 350).

Characteristic*a*	Measure
Growth measurements at study entry	
8-year-old boys (*n* = 297)	
Height (cm)	128.3 ± 5.9
Height *z*-score*b*	0.15 ± 1.02
Weight (kg)	26.4 ± 5.3
Weight *z*-score*b*	0.04 ± 1.25
BMI (kg/m^2^)	15.9 ± 2.2
BMI *z*-score*b*	–0.12 ± 1.28
Pubertal onset (genitalia stage ≥ 2)	65 (22)
9-year-old boys (*n* = 53)	
Height (cm)	132.7 ± 5.3
Height *z*-score*b*	–0.03 ± 0.88
Weight (kg)	27.9 ± 5.7
Weight *z*-score*b*	–0.27 ± 1.15
BMI (kg/m^2^)	15.8 ± 2.6
BMI *z*-score*b*	–0.42 ± 1.06
Pubertal onset (genitalia stage ≥ 2)	17 (32)
Birth and neonatal history (all boys, *n* = 350)	
Birth weight (kg)	3.4 ± 0.5
Gestational age (weeks)	39.0 ± 1.8
Duration of breast-feeding (weeks)	26.1 ± 33.9
Breast-fed	297 (85)
Maternal characteristics and exposures during pregnancy (all boys, *n* = 350)	
Mother’s age at son’s birth < 25 years	222 (64)
Mother overweight at study entry	132 (40)
Maternal alcohol consumption	48 (14)
Maternal tobacco smoking	25 (7)
Any household tobacco smoking	165 (48)
Maternal reported activities and exposures (all boys, *n* = 350)	
Ever employed at chemical plant	20 (6)
Herbicide/pesticide occupational exposure	5 (1)
Local gardening	210 (60)
Herbicide/pesticide personal use	309 (88)
BLL (µg/dL) at study entry (all boys, *n* = 350)	3.7 ± 2.5
Household characteristics (all boys, *n* = 350)	
Parental education (maximum)	
Secondary education or less	29 (8)
Junior college/technical training	198 (57)
University graduate	121 (35)
Household income reported at study entry (US$/month)	
< 175	107 (31)
175–250	88 (25)
> 250	154 (44)
Both parents living in the home at study entry	227 (65)
Data are mean ± SD, or *n* (%), unless stated otherwise. **a**Missing information: birth weight (*n* = 1), gestational age (*n* = 2), breast-fed (*n* = 5), mother’s age (*n* = 3), mother overweight (*n* = 21), mother drank during pregnancy (*n* = 11), mother smoked during pregnancy (*n* = 8), any household smoking (*n* = 6), mother employed at chemical plant (*n* = 10), pesticide occupational exposure (*n* = 12), local gardening (*n* = 6), pesticide/herbicide personal use (*n* = 9), parental education (*n* = 2), household income (*n* = 1). *b*WHO age-adjusted *z*-scores (WHO 2011).	

We compared boys with serum OCP measurements and those in the cohort without OCP measurements (data not shown) and found no significant differences in height, weight, BMI *z*-scores at study entry, or birth or family characteristics, except that more boys with OCP measurements had higher household income (69% high income vs. 54%, *p* = 0.001). Boys with OCP measurements had significantly lower total TEQs, DLCs, PCBs, and BLLs than did boys without OCP measurements. Mothers of boys with OCP measurements reported more personal pesticide use (91% vs. 77%, *p* < 0.001)

Median HCB and *p*,*p*´-DDE concentrations (Table 2) were 11 and 2.5 times higher, respectively, than the upper 95% confidence intervals (CIs) for the median values reported for 12- to 19-year-old U.S. adolescents in the 2003–2004 National Health and Nutrition Examination Survey. The median βHCH concentration in the same U.S. adolescent group was below the limit of detection and was not in the detectable range until the 95th percentile; thus, the Russian boys’ median βHCH level was almost 12 times higher than the upper 95% confidence limit of the 95th percentile for U.S. teens (Patterson et al. 2009). The medians (25th, 75th percentiles) for serum total TEQs, DLCs, and PCB concentrations were, respectively, 19.4 pg/g lipid (12.5, 29.2), 323 pg/g lipid (261, 416), and 208 ng/g lipid (151, 329). The median (25th, 75th percentiles) for BLL was 3.0 (2.0, 4.0) µg/dL. The OCPs were positively correlated with each other and the other organochlorine compounds, with the highest Spearman correlation (0.70) between βHCH and both total TEQs and PCBs. βHCH was also moderately correlated with *p*,*p*´-DDE (0.58) and HCB (0.54). BLL showed weaker correlations with the OCPs (0.10–0.24).

**Table 2:** Distribution of measured OCPs (ng/g lipid)*^a^* among 8- and 9-year-old boys enrolled in the Russian Children’s Study (*n* = 350).

Percentile								
OCP	*n*	10th	25th	50th (median)	75th	90th
HCB		350		80		107		159		247		365
βHCH		350		82		114		168		272		421
*p*,*p*´-DDE		350		122		189		287		492		866
**a**None below the limit of detection.												

**Figure 1:**
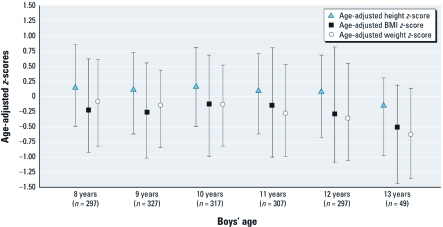
Median (25th, 75th percentiles) height, BMI, and weight *z*-scores over time among boys in the Russian Children’s Study (*n* = 350). Height and BMI *z*-scores are based on WHO (2011); weight *z*-scores, on CDC (2009).

*Growth measures at entry and during follow-up.* At study entry, most of the boys’ height, weight, and BMI (Table 1) were within the normal range according to child growth standards from WHO (de Onis et al. 2007) and CDC (McDowell et al. 2009). However, 18% of the boys were overweight (> 1 SD above the mean) (de Onis et al. 2007), 6% were underweight (defined as > 2 SD below the mean) (de Onis et al. 2007), and 2% of the boys’ heights were > 2 SD below the mean. During follow-up, the mean height, weight, and BMI *z*-scores remained relatively unchanged (Figure 1). The mean (± SD) HVs at 8–13 years of age were, respectively, 5.3 ± 0.8, 5.5 ± 0.8, 4.9 ± 1.0, 5.8 ± 1.8, and 6.7 ± 2.2 cm/year.

*Boys’ characteristics, household income, and BLLs as predictors of growth measures.* In multivariate models, for each 1-kg increase in birth weight, boys had significantly higher estimated *z*-scores (95% CI) for BMI and height of 0.65 (0.35, 0.94) and 0.63 (0.41, 0.85), respectively, over 4 years of follow-up. Boys from the lowest household income category had significantly lower estimated BMI *z*-score (95% CI) of –0.38 (–0.69, –0.07). Preterm birth was associated with significantly greater estimated height *z*-score (95% CI) over 4 years of follow-up of 0.59 (0.19, 0.98). High BLL was associated with significantly lower height *z*-score (95% CI) of –0.44 (–0.67, –0.21). However, BLL was not significantly associated with a change in BMI *z*-score.

**Figure 2:**
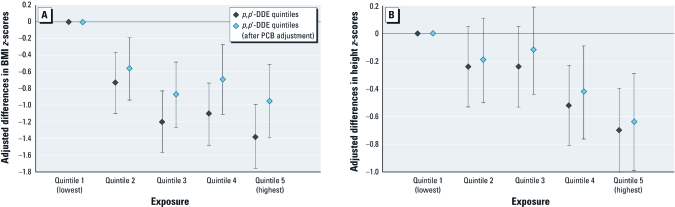
Adjusted differences in BMI *z*-scores (*A*) and height *z*-scores (*B*) between quintiles of serum *p*,*p*´-DDE versus lowest quintile, with and without adjustment for PCB quintiles, among boys in the Russian Children’s Study (*n* = 350).

*Multivariate associations of serum OCPs with growth measures.* In both univariate [see Supplemental Material, Table 1 (http://dx.doi.org/10.1289/ehp.1103743)] and multivariate models, boys with higher serum HCB, βHCH, and *p*,*p*´-DDE concentrations had lower mean BMI *z*-scores over 4 years of follow-up (Table 3, Figure 2A). At 12 years of age, the adjusted mean (95% CI) BMIs were 16.2 (15.5, 16.9) and 18.9 (18.0, 20.0) kg/m^2^ for the highest and lowest *p*,*p*´-DDE quintiles, respectively. The pattern for both HCB and βHCH showed a linearly decreasing trend over quintiles until the highest quintile, where there was either a plateau (βHCH) or a reversal (HCB) (Table 3). Sensitivity analyses including quintiles of TEQs, DLCs, or PCBs in the model had minimal impact on the OCP associations (data not shown), although including PCBs attenuated the OCPs’ associations with BMI *z*-scores (Figure 2A). In the model including all three OCPs, each individual estimate was attenuated, although significant associations of βHCH and *p*,*p*´-DDE with BMI *z*-scores remained (see Supplemental Material, Table 2).

**Table 3:** Associations of serum OCP with measures of growth over 4 years of follow-up in boys from the Russian Children’s Study*^a^* (*n* = 350).

HCB*b*			βHCH*c*			*p*,*p*´-DDE*d*		
Growth measure/quintile of exposure	Estimate (95% CI)	*p*-Value	Estimate (95% CI)	*p*-Value	Estimate (95% CI)	*p*-Value
Annual WHO age-adjusted BMI *z*-scores (*n* = 345)												
Quintile 1 (lowest)		Reference				Reference				Reference		
Quintile 2		–0.36 (–0.73, 0.02)		0.06		–0.61 (–0.98, –0.24)		0.001		–0.75 (–1.12, –0.38)		< 0.001
Quintile 3		–0.70 (–1.07, –0.32)		< 0.001		–1.09 (–1.46, –0.76)		< 0.001		–1.19 (–1.56, –0.82)		< 0.001
Quintile 4		–1.30 (–1.68, –0.91)		< 0.001		–1.33 (–1.70, –0.97)		< 0.001		–1.10 (–1.48, –0.72)		< 0.001
Quintile 5 (highest)		–0.84 (–1.23, –0.46)		< 0.001		–1.32 (–1.70, –0.95)		< 0.001		–1.37 (–1.75, –0.98)		< 0.001
Trend test				< 0.001				< 0.001				< 0.001
Annual WHO age-adjusted height *z*-scores (*n* = 345)												
Quintile 1 (lowest)		Reference				Reference				Reference		
Quintile 2		–0.25 (–0.55, 0.04)		0.09		–0.24 (–0.55, 0.06)		0.11		–0.25 (–0.53, 0.05)		0.09
Quintile 3		–0.04 (–0.33, 0.26)		0.81		–0.21 (–0.54, 0.08)		0.18		–0.24 (–0.53, 0.05)		0.10
Quintile 4		–0.33 (–0.63, –0.03)		0.03		–0.41 (–0.72, –0.12)		0.006		–0.52 (–0.81, –0.22)		< 0.001
Quintile 5 (highest)		–0.19 (–0.49, 0.11)		0.22		–0.28 (–0.59, 0.02)		0.08		–0.69 (–1.00, –0.39)		< 0.001
Trend test				0.18				0.03				< 0.001
Annual HV (*n* = 329)*e*												
Quintile 1 (lowest)		Reference				Reference				Reference		
Quintile 2		0.13 (–0.07, 0.33)		0.20		–0.06 (–0.26, 0.14)		0.57		–0.15 (–0.34, 0.06)		0.13
Quintile 3		0.06 (–0.14, 0.25)		0.60		0.05 (–0.16, 0.26)		0.64		–0.05 (–0.25, 0.16)		0.64
Quintile 4		–0.09 (–0.29, 0.11)		0.37		–0.16 (–0.36, 0.04)		0.13		–0.24 (–0.45, –0.04)		0.02
Quintile 5 (highest)		–0.05 (–0.25, 0.16)		0.66		–0.03 (–0.23, 0.18)		0.81		–0.22 (–0.43, –0.01)		0.04
Trend test				0.19				0.47				0.03
**a**Mixed-effects repeated measures regression model adjusted for age, birth weight, gestational age, household income, total calories consumed, percent calories from carbohydrate, protein, and fat, and BLL. **b**HCB quintiles (Q1–Q5, ng/g lipid): Q1, 31–98; Q2, 99–135; Q3, 136–184; Q4, 185–282; Q5, 283–2,660. **c**βHCH quintiles (Q1–Q5, ng/g lipid): Q1, 39–104; Q2, 105–144; Q3, 145–196; Q4, 197–302; Q5, 303–2,860. **d***p*,*p*´-DDE quintiles (Q1–Q5, ng/g lipid): Q1, 48–172; Q2, 173–246; Q3, 247–354; Q4, 355–549; Q5, 550–9,370. *e*Reduced number because at least two consecutive measures are required for calculation of change in height (e.g., HV).												

Similar to the univariate associations [see Supplemental Material, Table 1 (http://dx.doi.org/10.1289/ehp.1103743)], higher serum *p*,*p*´-DDE concentrations were associated with lower height *z*-scores over 4 years of follow-up, with a monotonic trend (Table 3). At 12 years of age, the adjusted mean (95% CI) heights were 146.8 (144.6, 149.1) and 151.5 (149.3, 153.7) cm for the highest and lowest *p*,*p*´-DDE quintiles, respectively. Adjustment for total TEQs and DLCs did not affect these associations (data not shown); however, adjusting for PCBs attenuated the associations between *p*,*p*´-DDE quintiles with height *z*-scores (Figure 2B). Height *z*-scores showed a significant decreasing trend with higher quintiles of βHCH, similar to the univariate associations (see Supplemental Material, Table 1), but this trend became nonsignificant after adjustment for other OCPs. Although in the univariate model higher serum HCB was associated with lower height *z*-scores (see Supplemental Material, Table 1), there was no consistent association in the multivariate model between HCB quintiles with height *z*-scores (Table 3). In the multiple OCP model, there was no change in the association between *p*,*p*´-DDE quintiles with height *z*-scores (see Supplemental Material, Table 2).

After adjustment for covariates, boys with the highest quintile of *p*,*p*´-DDE had a mean (95% CI) HV that was significantly decreased by –0.22 (–0.43, –0.01) cm/year over 4 years compared with the lowest quintile, with a nonlinear dose response (Table 3). After further adjustment for either pubertal stage or PCB quintiles, *p*,*p*´-DDE was no longer significantly associated with HV (data not shown). Neither serum HCB nor βHCH concentrations were associated with HV over 4 years of follow-up (Table 3).

In sensitivity analyses that included parental height and BMI and boy’s pubertal status, the associations were statistically significant and in the expected positive directions; however, the observed associations of OCPs with growth were not affected (data not shown); thus, we did not include these parental and pubertal status measures in the final models. We did not find evidence that age modified OCPs’ associations with growth (data not shown).

## Discussion

In the present study, Russian boys with higher peripubertal serum OCPs had lower age-adjusted BMI *z*-scores over 4 years of follow-up. These associations persisted after adjustment for serum DLCs, PCBs, and BLL. At 12 years of age, the difference in estimated BMI was 2.7 kg/m^2^ lower among boys in the highest than in the lowest *p*,*p*´-DDE quintile. In addition, higher serum *p*,*p*´-DDE was associated with lower height *z*-scores over 4 years, independent of serum DLCs, PCBs, and lead. At 12 years of age, the difference in estimated height was 4.7 cm lower for boys in the highest than in the lowest *p*,*p*´-DDE quintile.

During pubertal maturation, especially among boys, interference with thyroid hormones, insulin-like growth factor 1 (IGF-1), and testosterone signaling may affect linear growth and weight gain. In animal studies, DDE was shown to act as an antiandrogen (Kelce and Wilson 1997). In a study among Spanish children, DDE was associated with lower serum IGF-1 (Zumbado et al. 2010). In several human studies, DDE, βHCH, and HCB were associated with thyroid hormone disruption (Pearce and Braverman 2009).

Most prior epidemiologic studies have not found an association of serum DDT or DDE with height (Cupul-Uicab et al. 2010; Dhooge et al. 2010; Gladen et al. 2004; Jusko et al. 2006; Karmaus et al. 2009; Pan et al. 2010; Verhulst et al. 2009). All of these studies, with one exception (Dhooge et al. 2010), measured a different window of exposure, that is, prenatal or lactational exposure to DDT or DDE. Although studies using historical (1959–1967) (Gladen et al. 2004; Jusko et al. 2006) or agricultural (Cupul-Uicab et al. 2010) exposures had higher DDE exposures than ours, others were conducted after DDT was banned, so exposure levels were lower than our cohort. Many of these studies followed their cohorts only through the prepubertal period and did not extend follow-up through the adrenarchal hormone–mediated increase in weight or the sex steroid–induced pubertal growth spurt (Cupul-Uicab et al. 2010; Jusko et al. 2006; Pan et al. 2010; Verhulst et al. 2009), which may partially contribute to their null findings. However, two studies found associations of DDE with reduced height. A prospective U.S. study (*n* = 1,712) observed an association of DDE concentrations with shorter height through 7 years of age based on stored prenatal serum samples collected during a period of DDT use (1959–1966) (Ribas-Fito et al. 2006). A German study (*n* = 343) using estimated early childhood serum DDE concentrations and both prospective and retrospective growth data found higher serum DDE associated with shorter height in 8-year-old girls (Karmaus et al. 2002).

In multivariate analysis, we found higher serum βHCH concentrations associated with lower height *z*-scores over 4 years of follow-up. However, after adjusting for all serum OCPs, the association was no longer significant. To our knowledge, only one previous small (*n* = 12) study of hospitalized children in the Aral Sea region examined, and did not find, a relationship of serum βHCH with childhood linear growth (Mazhitova et al. 1998).

We observed significant negative associations for all OCPs with lower BMI *z*-scores over 4 years of follow-up. Two cross-sectional studies found associations of childhood serum DDE (Mazhitova et al. 1998) and HCB (a Flemish cohort, *n* = 1,679; Dhooge et al. 2010) with lower BMI. However, most of the published literature concerns studies of prenatal or lactational OCP exposure, compared with our peripubertal measurements, and found either positive associations between prenatal OCP concentrations and childhood BMI (Gladen et al. 2000; Mendez et al. 2011; Smink et al. 2008; Verhulst et al. 2009) and adult weight (Karmaus et al. 2009) or null associations between prenatal (Cupul-Uicab et al. 2010; Gladen et al. 2004; Jusko et al. 2006) or lactational (Gladen et al. 2000; Pan et al. 2010) OCP concentrations and BMI. Populations in studies that reported null associations of prenatal DDE with BMI had higher levels than our peripubertal DDE concentrations. In those studies, their lowest prenatal DDE categories were comparable to the highest quintile in our cohort. However, most studies that reported positive associations between prenatal OCPs and BMI had lower OCP concentrations than our cohort, comparable to our lower quintiles of exposure. Interestingly, Mendez et al. (2011) reported positive associations between BMI and DDE concentrations up to 750 ng/g lipid but observed a decrease in BMI when DDE concentrations exceeded 750 ng/g lipid, comparable to the median in our highest DDE quintile. In our cohort, we did not find any evidence of a nonlinear dose–response relationship between DDE and BMI *z*-scores. These contradictory results across studies suggest that range and timing of exposure may be important factors for OCPs’ association with BMI.

An alternative explanation for associations of higher serum OCP concentrations with lower BMI *z*-scores is that serum OCPs may be lower in those with a larger body mass because of a greater volume of distribution and sequestration in adipose tissue. A dilutional effect of increased growth (Wolff et al. 2005) on serum OCP concentrations would indicate reverse causation whereby BMI led to a decrease in OCP concentrations, rather than a causative effect of OCPs on BMI. The reverse causation hypothesis may be consistent with the stronger associations observed between OCPs and BMI *z*-scores than between OCP and height *z*-scores. However, these differences may also reflect differences in biological effects of the individual OCPs on BMI and height, rather than dilutional effects. If prenatal and early life exposures, through placental transfer and breast-feeding, were the primary source of OCP exposure, then dilutional effects would be a more plausible explanation for the associations we observed. However, in our cohort there is ongoing OCP exposure from local soil and foods (Ecological Analytical Center 2007). It was not possible to estimate the contributions from these sources to the boys’ serum concentrations. In the present analysis, we cannot determine whether associations between serum OCPs and BMI *z*-scores were attributable to differences in body composition, such as reduced lean muscle mass versus body fat, because BMI is a crude approximation of body fat. Prenatal exposure to dioxins and furans was not associated with childhood weight among the Yu-Cheng cohort but was significantly associated with reduced lean muscle mass (Guo et al. 1994). Therefore, alterations in the ratio of lean muscle mass and body fat may result from exposure to some organochlorine compounds. In future analyses, we will examine whether OCPs are associated with alterations in body composition using longitudinal skin fold and bioelectric impedance data.

Limitations of our study were that we did not have measures of prenatal OCPs and may have missed a critical window for OCPs’ effect on growth, although findings on prenatal exposures have been inconsistent (Eskenazi et al. 2009; Mendez et al. 2011; Smink et al. 2008). Moreover, our childhood measures of serum OCPs may be a surrogate for prenatal exposure because childhood levels of lipophilic compounds often track closely with prenatal levels (Patandin et al. 1999), especially in a primarily breast-fed population such as ours. Also, there was likely continuing environmental exposure to these compounds in this community; thus, the boys’ serum concentrations reflect both pre- and postnatal exposures.

Our results from the Russian Children’s Study provide evidence that OCPs affect children’s growth during the critical peripubertal period. These compounds, especially *p*,*p*´-DDE, were associated with lower height and BMI *z*-scores, even after adjustment for other environmental exposures and known predictors. Childhood exposure to OCPs, especially DDE, is still a public health concern because of their environmental persistence and the continued use of DDT in some countries. OCPs may affect children’s growth by affecting hormones associated with growth (Kelce and Wilson 1997; Li et al. 2008; Pearce and Braverman 2009; Zumbado et al. 2010) and body composition (Guo et al. 1994; Smink et al. 2008). Our future research in Chapaevsk will examine whether these compounds are associated with alterations in the ratio of fat to muscle mass and growth-associated hormones.

## Supplemental Material

(98 KB) PDF
