# Supplementary material for: Serum Concentrations of Organochlorine Pesticides and Growth among Russian Boys
Source: Environ Health Perspect. 2011 Oct 7;120(2):303–8. doi: 10.1289/ehp.1103743 (PMC3279441; doi:10.1289/ehp.1103743)
Supplement: (98 KB) PDF [file ehp.1103743.s001.pdf]

Supplemental Material

Serum Levels of Organochlorine Pesticides and Growth among Russian Boys

Jane S. Burns[1], Paige L. Williams[2], Oleg Sergeyev[3,4], Susan A. Korrick[1,5], Mary M. Lee[6], Boris Revich[7], Larisa Altshul[8,9], Julie T. Del Prato[1], Olivier Humblet[10], Donald G. Patterson Jr.[11,12,13,14,15], Wayman E. Turner[11], Mikhail Starovoytov[16], Russ Hauser[1]

[1]Environmental and Occupational Medicine and Epidemiology Program, Department of Environmental Health, Harvard School of Public Health, Boston, Massachusetts, USA

[2]Department of Biostatistics, Harvard School of Public Health, Boston, Massachusetts, USA

[3]Department of Physical Education and Health, Samara State Medical University, Samara, Russia

[4]Chapaevsk Medical Association, Chapaevsk, Russia

[5]Channing Laboratory, Department of Medicine, Brigham and Women's Hospital, Harvard Medical School, Boston, Massachusetts, USA

[6]Pediatric Endocrine Division, Departments of Pediatrics and Cell Biology, University of Massachusetts Medical School, Worcester, Massachusetts, USA

[7]Department of Environmental Health, Institute for Forecasting, Russian Academy of Sciences, Moscow, Russia

[8]Environmental Health and Engineering, Inc., Needham, Massachusetts, USA

[9]Exposure, Epidemiology, and Risk Program, Department of Environmental Health, Harvard School of Public Health, Boston, Massachusetts, USA

[10]Division of Immunology and Allergy, Department of Pediatrics, Stanford University, Stanford, California, USA

[11]Centers for Disease Control and Prevention, Atlanta, Georgia, USA

[12]EnviroSolutions Consulting, Inc., Auburn, Georgia, USA

[13]Axys Analytical Solutions, Sidney, British Columbia, Canada

[14]Fluid Management Systems, Boston, Massachusetts, USA

[15]Exponent, Inc., Maynard, Massachusetts, USA

[16]Russian Institute of Nutrition, Moscow, Russia

Table of Contents:

Supplemental Table 1

Supplemental Table 2

Supplemental Material, Table 1. Univariate associations of serum organochlorine pesticides with measures of growth over 4 years of follow-up in boys from the Russian Children's Study[a] (N=350)

	HCB[b]		βHCH[c]		p,p´-DDE[d]	
Growth measure/ quintiles of exposure	Estimate (95% CI)	*p*-Value	Estimate (95% CI)	*p*-Value	Estimate (95% CI)	*p*-Value
Annual WHO age-adjusted BMI z-scores (*n* = 345)						
Quintile 1 (lowest)	Reference		Reference		Reference	
Quintile 2	-0.32 (-0.71, 0.07)	0.11	-0.51 (-0.89, -0.13)	0.008	-0.67 (-1.06, -0.29)	<0.001
Quintile 3	-0.73 (-1.12, -0.35)	<0.001	-1.08 (-1.46, -0.70)	<0.001	-1.06 (-1.45, -0.68)	<0.001
Quintile 4	-1.27 (-1.66, -0.88)	<0.001	-1.37 (-1.75, -1.00)	<0.001	-1.15 (-1.54, -0.76)	<0.001
Quintile 5 (highest)	-0.95 (-1.34, -0.57)	<0.001	-1.30 (-1.68, -0.93)	<0.001	-1.34 (-1.72, -0.95)	<0.001
Trend test		<0.001		<0.001		<0.001
Annual WHO age-adjusted height z-scores (*n* = 345)						
Quintile 1 (lowest)	Reference		Reference		Reference	
Quintile 2	-0.25 (-0.58, 0.07)	0.13	-0.28 (-0.60, 0.04)	0.08	-0.25 (-0.57, 0.06)	0.12
Quintile 3	-0.11 (-0.43, 0.22)	0.52	-0.28 (-0.60, 0.05)	0.09	-0.19 (-0.50, 0.13)	0.25
Quintile 4	-0.39 (-0.71, 0.06)	0.02	-0.59 (-0.91, -0.28)	<0.001	-0.59 (-0.91, -0.28)	<0.001
Quintile 5 (highest)	-0.37 (-0.69, 0.05)	0.02	-0.43 (-0.75, -0.10)	0.01	-0.78 (-1.09, -0.46)	<0.001
Trend test		0.02		0.001		<0.001
Annual height velocity (*n* =329)[e]						
Quintile 1 (lowest)	Reference		Reference		Reference	
Quintile 2	0.10 (-0.10, 0.30)	0.32	-0.08 (-0.28, 0.12)	0.41	-0.14 (-0.34, 0.06)	0.18
Quintile 3	0.03 (-0.17, 0.22)	0.78	0.03 (-0.17, 0.23)	0.76	-0.02 (-0.23, 0.18)	0.82
Quintile 4	-0.13 (-0.33, 0.07)	0.20	-0.22 (-0.42, -0.03)	0.03	-0.22 (-0.42, -0.02)	0.03
Quintile 5 (highest)	-0.10 (-0.30, 0.11)	0.35	-0.07 (-0.27, 0.13)	0.50	-0.23 (-0.43, -0.03)	0.02
Trend test		0.07		0.20		0.02

[a]Mixed effects repeated measures regression model without adjustment for other covariates.

[b]HCB quintiles (Q1-Q5, ng/g lipid): Q1 31 – 98; Q2 99 -135; Q3 136 – 184; Q4 185 - 282; Q5 283 – 2660;

[c]βHCH quintiles (Q1-Q5, ng/g lipid): Q1 39 – 104; Q2 105 – 144; Q3 145 – 196; Q4 197 – 302; Q5 303 – 2860;

[d]p,p´-DDE quintiles (Q1-Q5, ng/g lipid): Q1 48 – 172; Q2 173 – 246; Q3 247 – 354; Q4 355 – 549; Q5 550 – 9370

[e]Reduced number since at least 2 consecutive measures are required for calculation of change in height (e.g. height velocity).

Supplemental Material, Table 2: Associations of serum organochlorine pesticides (OCPs) with measures of growth over 4 years of follow-up in boys from the Russian Children's Study[a] including all OCPs simultaneously (N=350)

	HCB[b]		βHCH[c]		p,p´-DDE[d]	
Growth measure/ quintile of exposure	Estimate (95% CI)	*p*-Value	Estimate (95% CI)	*p*-Value	Estimate (95% CI)	*p*-Value
Annual WHO age-adjusted BMI z-scores (*n* = 345)						
Quintile 1 (lowest)	Reference		Reference		Reference	
Quintile 2	-0.06 (-0.42, 0.30)	0.74	-0.30 (-0.67, 0.07)	0.11	-0.41 (-0.78, -0.05)	0.03
Quintile 3	-0.30 (-0.67, 0.07)	0.11	-0.72 (-1.12, -0.32)	<0.001	-0.80 (-1.18, -0.43)	<0.001
Quintile 4	-0.73 (-1.13, -0.33)	<0.001	-0.72 (-1.15, -0.29)	0.001	-0.48 (-0.88, -0.07)	0.02
Quintile 5 (highest)	-0.26 (-0.68, 0.15)	0. 21	-0.70 (-1.17, -0.22)	0.004	-0.74 (-1.17, -0.30)	0.001
Trend test		0.02		<0.001		0.002
Annual WHO age-adjusted height z-scores (*n* = 345)						
Quintile 1 (lowest)	Reference		Reference		Reference	
Quintile 2	-0.22 (-0.52, 0.08)	0.50	-0.06 (-0.37, 0.25)	0.69	-0.21 (-0.51, 0.10)	0.18
Quintile 3	-0.04 (-0.27, 0.34)	0.35	-0.001 (-0.33, 0.33)	0.99	-0.22 (-0.54, 0.09)	0.17
Quintile 4	-0.16 (-0.49, 0.18)	0.81	-0.08 (-0.44, -.28)	0.67	-0.50 (-0.83, -0.15)	0.005
Quintile 5 (highest)	-0.12 (-0.46. 0.23)	0.15	-0.19 (-0.21, 0.58)	0.36	-0.73 (-1.10, -0.37)	<0.001
Trend test		0.69		0.45		<0.001
Annual height velocity (*n* =329)[e]						
Quintile 1 (lowest)	Reference		Reference		Reference	
Quintile 2	0.11 (-0.10, 0.31)	0.33	-0.02 (-0.23, 0.19)	0.89	-0.14 (-0.35, 0.07)	0.19
Quintile 3	0.04 (-0.17, 0.25)	0.72	0.11 (-0.12, 0.34)	0.33	-0.05 (-0.27, 0.16)	0.64
Quintile 4	-0.06 (-0.29, 0.17)	0.59	0.003 (-0.24, 0.25)	0.11	-0.23 (-0.47, 0.00)	0.05
Quintile 5 (highest)	-0.08 (-0.31, 0.16)	0.51	0.17 (-0.11, 0.44)	0.22	-0.27 (-0.52, -0.02)	0.03
Trend test		0.26		0.26		0.04

[a]Mixed effects repeated measures regression model adjusted for age, birthweight, gestational age, household income, total calories consumed, percent calories from carbohydrate, protein, and fat, BLL, *and all OCPs*.

[b]HCB quintiles (Q1-Q5, ng/g lipid): Q1 31 – 98; Q2 99 -135; Q3 136 – 184; Q4 185 - 282; Q5 283 – 2660;

[c]βHCH quintiles (Q1-Q5, ng/g lipid): Q1 39 – 104; Q2 105 – 144; Q3 145 – 196; Q4 197 – 302; Q5 303 – 2860;
[d]p,p´-DDE quintiles (Q1-Q5, ng/g lipid): Q1 48 – 172; Q2 173 – 246; Q3 247 – 354; Q4 355 – 549; Q5 550 – 9370

[e]Reduced number since at least 2 consecutive measures are required for calculation of change in height (e.g. height velocity).
